# Vitamin D, Sunlight and Prostate Cancer Risk

**DOI:** 10.4061/2011/281863

**Published:** 2011-06-08

**Authors:** Krishna Vanaja Donkena, Charles Y. F. Young

**Affiliations:** ^1^Department of Biochemistry and Molecular Biology, Mayo Clinic College of Medicine, Rochester, MN 55905, USA; ^2^Departments of Urology, Biochemistry and Molecular Biology, Mayo Clinic College of Medicine, Rochester, MN 55905, USA

## Abstract

Prostate cancer is the second common cancer in men worldwide. The prevention of prostate cancer remains a challenge to researchers and clinicians. Here, we review the relationship of vitamin D and sunlight to prostate cancer risk. Ultraviolet radiation of the sunlight is the main stimulator for vitamin D production in humans. Vitamin D's antiprostate cancer activities may be involved in the actions through the pathways mediated by vitamin D metabolites, vitamin D metabolizing enzymes, vitamin D receptor (VDR), and VDR-regulated genes. Although laboratory studies including the use of animal models have shown that vitamin D has antiprostate cancer properties, whether it can effectively prevent the development and/or progression of prostate cancer in humans remains to be inconclusive and an intensively studied subject. This review will provide up-to-date information regarding the recent outcomes of laboratory and epidemiology studies on the effects of vitamin D on prostate cancer prevention.

## 1. Introduction

The World Health Organization (http://globocan.iarc.fr/factsheets/cancers/prostate.asp) indicates that prostate cancer is the second most frequently diagnosed cancer in men (903,000 new cases) and has about 258,000 deaths of this cancer worldwide in 2008. The highest incident rates are among the countries of Australia/New Zealand Western, Northern Europe, and Northern America, and the lowest age-matched incidence rates are those in South-central Asia. In the USA alone, the American Cancer Society (http://www.cancer.org/Cancer/ProstateCancer/DetailedGuide/prostate-cancer-key-statistics) estimated death and newly diagnosed cases of prostate cancer were 32,050 and 217,730 men, respectively, in year 2010. Moreover, in USA, the total medical expenditure for prostate cancer treatment was estimated as $1.3 billion in year 2000, which represents a 30% increase compared to that in 1994. In year 2004, 2.3 billion was estimated for prostate cancer alone [1]. As being a prevalent cancer disease in men, current total cost for PCa prostate cancer treatments in USA would much exceed $2.3 billion. 

Until now, etiology of prostate cancer is still largely unknown. However, it has been suggested that there are several potential risk factors that may change incidence rates of this cancer, including diet/nutrition, physical activities, and others [2, 3]. Epidemiologic and laboratory studies in nutrition and diet as modifiable risk factors seem to build strong concepts of cancer chemoprevention, a strategy seeking the reduction of cancer risk by the use of chemical agents [3–7]. Conceptually, these agents may be used to prevent, delay, or reverse cancer formation as well as progression. Indeed, due to its long latency of disease onset and high incidence and mortality rates, prostate cancer should be an ideal target for chemoprevention. Proper diets may eventually reduce 50–60% incidence of prostate cancer and many other cancers. Therefore, the potential impact of prostate cancer chemoprevention could be enormous, with respect to prostate cancer patients, in saving life, increasing quality of life and reducing community financial burden.

One such dietary factor having anticancer properties is vitamin D. Vitamin D is very important for normal physiology [8, 9]. The natural way to obtain vitamin D in the body is by exposing skin to sunlight [9, 10]. Avoiding skin carcinogenesis and with many other reasons, skins may not receive sufficient amounts of sunlight exposure for producing enough vitamin D; fortified vitamin D in some commonly consumed foods or supplement forms has been used for human health purposes. Evidence suggests that vitamin D in our body may be negatively associated with the development and/or progression of several cancers including prostate cancer [10, 11].As discussed below, although experimental results from *in vitro* and preclinical models showed strong support of antiprostate cancer activities of this vitamin, epidemiological studies and clinical trials on human subjects hardly produce unanimous agreements for the potential of antiprostate cancer efficacy. In this article, we will present recent findings of laboratory results in supporting of vitamin D's antiprostate cancer effects and discuss conflicting epidemiological findings of the vitamin in human subjects.

## 2. Vitamin D Metabolism

Although the previtamin D 7-dehydrocholesterol was thought to be produced in the gut wall cells and transported to skin cells, actually skin cells can synthesize their own 7-dehydrocholesterol, which in turn is converted to a provitamin D, cholecalciferol, or vitamin D_3_, by isomerization upon ultraviolet B (UVB) radiation of sunlight in epidermis [12–14]. Further photoreaction of vitamin D_3_ by UVB absorption may generate inactive metabolites. Vitamin D_3_ is metabolized to calcidiol 25(OH)D_3_ in the liver by the mitochondrial sterol 27-hydroxylase (27-hydroxylase; (CYP27A1) and converted to a biologically active vitamin D, calcitriol/1,25-dihydroxyvitamin D_3_ (1,25(OH)_2_D_3_), by 1*α*-hydroxylase (CYP27B1) in the kidney and other tissues including the prostate [15–18]. Usually circulating 25(OH)D_3_ level is used to determine vitamin D nutritional status, because it is a predominant form of vitamin D in blood stream and has a much longer half life than that of 1,25(OH)_2_D_3_ (i.e., 15 days versus 15 hours) [19]. Importantly, its serum concentrations may be correlated with total vitamin D levels from both endogenous production and dietary uptakes [20–23]. However, 25(OH)D_3_ is by no means a perfect marker for active vitaminD_3_status.For example, it is questionable whether measuring 25(OH)D_3_ can represent the bioavailability of vitamin D_2_ (ergocalciferol, a vitamin D proform derived from fungus products) versus vitamin D_3_. Some studies [24, 25] but not other [26] showed that vitamin D_3_ supplementation could increase 25(OH)D_3_ to higher levels than the use of vitamin D_2_. In fact, blood 25(OH)D_3_ levels can be affected by substrate availability through adiposity sequestration, skin pigmentation, physical activity [20, 27, 28], and the consumption of dietary factors such as genistein and folate [29–32].

As indicated above, 25-hydroxyvitamin D_3_ 1-*α* hydroxylase or 1-*α* hydroxylase (CYP27B1) is also expressed in the prostate, meaning that prostate cells can produce the active form of vitamin D_3_. This enzyme activity has been demonstrated in human primary prostatic cell cultures as well as prostate cancerous cell lines. Obviously, this enzyme may have a role in negatively regulating prostate cell proliferation [33]. Human prostatic cancerous cells seem to have reduced activity or expression levels of 1-*α* hydroxylase compared to normal or benign prostatic cells, therefore, losing ability to synthesize 1,25(OH)_2_D_3_ [34]. 

There are not many studies demonstrating intraprostatic concentrations of vitamin D metabolites. One report showed that prostatic 1,25(OH)_2_D_3_ levels were higher than that in blood circulation in domestic pigs [35]. Other study found that within 24 hours of intravenous injection of 1,25-dihydroxyvitamin D_3,_ less than 1% of the vitamin D in blood was detected in rat prostate tissues [36]. The third study also demonstrated the potential intraprostatic vitamin D metabolism in human prostate [37]. 25OHD_3_, 24,25(OH)_2_D_3_, and 1,25(OH)_2_D_3_ were all detected in prostate tissues obtained by prostatectomy. This particular study with a very small sample size seemed to suggest that levels of 24,25(OH)_2_D_3_ and 1,25(OH)_2_D_3_ in the prostate were higher than in serum tested.

One possible mechanism for the reduced expression of 1-*α* hydroxylase may be due to hypermethylation or repressive histone modification of its promoter, which could implicate prostate cancer development and progression [38–40]. Other possibility includes posttranslational suppression of enzymatic activity [39, 41]. 

It was reported that 25(OH)D_3_ but not 1,25(OH)_2_D_3_ can enhance the expression of 1-*α* hydroxylase in cultured prostatic cells [42]. Because of this, the authors of the studies suggested that high concentrations of 25(OH)D_3_ might be used as antiprostate cancer agent instead of large doses of 1,25(OH)_2_D_3_ to avoid hypercalcemia side-effects. 

Opposed to 1-*α* hydroxylase, 25-hydroxyvitamin D_3_ 24-hydroxylase (CYP24A1) is a catabolic enzyme causing inactivation of 1,25(OH)_2_D_3_ that might implicate resistance to antiproliferation effects of 1,25(OH)_2_D_3_ [43, 44]. However, some studies suggested that this 24-hydroxylase is downregulated in prostate tumor cells [45]. By examining 30 paired human prostate benign and primary malignant tissues and three prostate cancer cell lines, the study demonstrated that a significant number of malignant tissues had lower mRNA expression and higher promoter methylation levels of the 24-hydroxylase compared to those of benign tissues. In addition, two out of three cancer cell lines tested had high methylation and low expression levels of the enzyme gene. In these two cell lines, that is, PC-3 and LNCaP, treatments with the DNA methyltransferase inhibitor 5-aza-2′-deoxycytidine and/or the inhibitor of histone deacetylases trichostatin A can activate the expression of this gene, suggesting that promoter DNA methylation, and repressive histone modifications play roles in repressing its expression [45]. Intriguingly, it has been shown that 1,25(OH)_2_D_3_ can induce the expression of the 24-hydroxylase in PC-3, LNCaP, DU145, and primary prostatic stromal cells, perhaps through (VDR) to bind a VDR responsive element (VDRE) [43–45]. A recent study found that a genetic single nucleotide polymorphism in the VDRE of the 24-hydroxylase promoter could reduce the expression and activity of this enzyme [46]. In addition, 1,25(OH)_2_D_3_ may modulate the expression of alternative splicing forms of the 24-hydroxylase in prostate cancer cells [47]. The significance of the splicing forms in prostate cells remains unclear. Not surprisingly, the 24-hydroxylase activity in prostate cancer cells could be inversely related to inhibitory proliferation effects of 1,25(OH)_2_D_3_ [44]. It was reported that the androgen dihydrotestosterone was able to inhibit the inducible effect of 1,25(OH)_2_D_3_ on 24-hydroxylase expression and activity in prostate cancer cells [48, 49]. This seems to indicate that a cross-talk of androgen receptor and VDR was at work. Furthermore, the same group of the authors showed that by suppressing the 24-hydroxylase androgens can largely increase antiproliferative effects of 1,25(OH)_2_D_3_. Since prostate stroma may provide an important microenvironment for prostate cancer development, these authors also showed that retinoic acid via retinoic acid receptor alpha inhibited the 24-hydroxylase expression in human prostatic stromal cells P29SN and P32S [50]. When treated with both retinoic acid and 1,25(OH)_2_D_3_. synergistic growth inhibitory effects were observed in these cells. 

Thus the above studies clearly demonstrated that the 24-hydroxylase is a useful target for increasing anticancer efficacy of vitamin D. Genistein, a soy isoflavone, was shown to be capable of enhancing antiproliferative effect of 1,25(OH)_2_D_3_ on DU145 cells by repressing the expression of the 24-hydroxylase [51, 52]. Moreover, genistein in nanomolar concentrations was able to inhibit enzymatic activity of the 24-hydroxylase as well as to upregulate the expression of VDR. Recent studies [53, 54] reported that a nonspecific, broad inhibitor of cytochrome P450 enzymes, ketoconazole or a specific 24-hydroxylase inhibitor, RC2204 was used in PC-3 cell culture or xenograft, respectively, to demonstrate that they can suppress 24-hydroxylase activities and enhance antitumor growth potency of 1,25(OH)_2_D_3_.

## 3. Action of VDR in Prostate Cancer Cells

Anticancer activities of vitamin D have been suggested to act mainly through its nuclear receptor or VDR. The VDR is a member of nuclear receptor super family, whose functions act as ligand-dependent transcription factor in the nucleus [54–56]. In addition, upon ligand activation, this receptor requires to form a heterodimer with the retinoid X receptor (RXR) in order to bind a specific genomic DNA sequence, that is, a VDRE to activate or repress gene expression [54–56]. RXR of the VDR heterodimer may be subjected to phosphorylation by prolonged activation of the mitogen-activated protein kinase pathway, resulting in the impairment of VDR-mediated prostate cell growth inhibition effects [57]. Recently, a report showed that the vitamin D receptor can form a heterodimer with retinoic acid receptor gamma [58]. Although it has been shown that androgen receptor and VDR may cross talk each other in their pathways [49, 59, 60], the two receptors probably do not have direct interactions. It has been reported that the expression of VDR can be regulated by several hormones including androgens, vitamin D, parathyroid hormone, retinoic acid, and glucocorticoids [55, 56]. However, the regulation of the basal line expression of the receptor is not well studied. Besides, the genomic gene regulation effects, through a so-called nongenomic mechanism, the same receptor activated by vitamin D in the plasma membrane may also have rapid modulation effects on cellular functions [55, 56, 61]. Both genomic and nongenomic effects of VDR have been demonstrated in prostate cells [43, 62, 63]. The question also arises if there is a separate membrane VDR. According to other investigators [64–66], protein disulfide isomerase family A, member 3 (PDIA3) has been identified as a membrane associated, 1,25(OH)_2_D_3_ binding protein/receptor that may exhibit some rapid nongenomic actions of 1,25(OH)_2_D_3_. PDIA3 with 1,25(OH)_2_D_3_ binding ability has recently been detected in several human prostate cell lines [66]. The significance of PDIA3-mediated 1,25(OH)_2_D_3_ action in prostate cancer cells requires further studies. 

Vitamin D mainly via VDR's genomic effects may suppress prostate cancer cellular dysfunctions including the inhibition of cell proliferation, cell cycle progression, cell invasiveness, angiogenesis, or induction of cell differentiation and apoptosis [67, 68]. These prostate cancer cellular functions can be altered by the ability of ligand-activated VDR to change the expression and/or functions of many downstream key genes, for example, decrease of c-Myc [69, 70], telomerase [71], *BCL-2* [72], *α*6 and *β*4 integrins [73], cyclin-dependent kinase 2 (C*DK2)* activity [74], and phosphorylation of the retinoblastoma protein [75], and increase of the (CDK) inhibitors *p21* ^Waf/Cip1^ and *p27* ^Kipl^ [74, 76–78] and growth arrest and DNA damage-inducible gene gamma (GADD45*γ*) [79]. In addition, active vitamin D_3_ and its analogs may increase the expression of E-cadherin [78] and the activity of tissue inhibitor of metalloproteinase-1 (TIMP-1) as well as decrease the expression and activity of MMP-9 [80], thereby decreasing invasive and metastatic potentials of prostate cancer cells studied.

Many angiogenic and proinflammatory regulators may play crucial roles in prostate tumorigenesis and progression [81–84]. It has been shown that 1*α*,25-dihydroxyvitamin D_3_ [1,25(OH)_2_D_3_] inhibits tumor angiogenesis *in vitro* and *in vivo* [85]. Interleukin 6 (IL-6) is one such molecule that may implicate in prostate cancer progression. Calcitriol was shown to inhibit tumor necrosis factor *α* mediated increase of IL-6 in primary prostate cells [86]. Another pro-inflammatory cytokine interleukin 8 (IL-8) may also have angiogenic and tumorigenic potentials in prostate cancer [83]. Calcitriol can lower the IL-8 levels in two immortalized human prostate epithelial cell lines (HPr-1 and RWPE-1) and three prostate cancer cell lines (i.e., LNCaP, PC-3, and DU145) [87], by reducing NF*κ*B p65 nuclear translocation and gene transcription of IL-8. Calcitriol may have radiosensitization effects on prostate cancer cells by suppressing ion radiation-mediated activation of the NF*κ*B related RelB, which subsequently reduces the transcription of manganese superoxide dismutase (MnSOD) [88]. Increased antioxidant activities of MnSOD can cause radiation resistant. It was reported that calcitriol reduces the protein and mRNA expressions of both the hypoxia-inducible factor (HIF)-1*α* subunit and the vascular endothelial growth factor (VEGF) in several human cancer cells including prostate cancer cells under hypoxia conditions [89]. Furthermore, using transgenic adenocarcinoma of the mouse prostate (TRAMP)-2 tumors transplanted into either wild-type or VDR knockout (KO) mice with calcitriol treatments, it was found that tumors in the KO mice were larger than that in wild-type mice, suggesting ligand-induced VDR growth inhibitory effects in wild-type mice. Similarly, enlarged blood vessels and increased vessel volume in TRAMP-2 tumors were found in the VDR knockout mice, suggesting that antitumor angiogenesis was directly affected through VDR and calcitriol at tumor sites. HIF-1*α*, VEGF, angiopoietin 1, and platelet-derived growth factor-BB levels were increased in tumors from KO mice [90]. The importance of VDR in negatively regulating prostate cancer progression is further confirmed in the LPB-Tag model of prostate in VDR knockout versus VDR wild-type mice [60]. 

Vitamin D may also influence genes in the metabolism of prostaglandins (PGs) that can induce the inhibition of the expression of the PG synthesizing cyclooxygenase-2 (COX-2) and the PG receptors EP2 and FP, and increased expression of PG inactivating 15-prostaglandin dehydrogenase [67, 91–93]. The alteration of these gene expressions would decrease the cell proliferative stimulus of PGs in prostate cancer cells.

## 4. Sunlight Exposure and Prostate Cancer 

Vitamin D deficiency or insufficiency has become a public health concern in large proportions of the populations in the United States and Northern European countries especially among ethnic groups with dark skin, and others such as those with physical inactive and little sun exposure. As mentioned above, sunlight exposure may increase vitamin D synthesis in the skin which has been thought to be beneficial to protect from some types of cancer, including prostate cancer. Of course, prolonged sunlight or UVB exposure without adequate skin protection can cause skin cancer. Indeed, there are many ecological and observational studies including case-control and prospective studies [94–106] showing a high degree of consistent results that sunlight exposure is inversely associated with prostate cancer risk.Geographic regions with less sunlight exposure seem to be related to an increased prostate cancer mortality [96, 97]. Studies [102, 103] also showed that patients diagnosed with prostate cancer in summer may have higher survival rates than that of patients in the winter due to seasonal UV irradiance levels. There are epidemiological studies [101, 107–109] suggesting that the ethnic groups with dark skin could be associated with high prostate cancer risk because high skin pigments may reduce the absorption of UV radiation. However, a study reported that black men did not increase their prostate cancer risk in terms of sunlight exposure when compared to white counterparts [110]. There is epidemiological evidence that shows skin cancer patients may have reduced risk for procuring certain types of secondary cancer including prostate cancer [111–113]. However, the result of a study did not support the notion that sunlight induced skin cancer can protect against prostate cancer risk [114]. Although there are overwhelming number of studies indicating that UVB exposure from sunlight consistently reduce risk of prostate cancer development and progression, yet not every study fully supports this idea. For instance, a population-based nested case-control study and meta-analysis [115] only provided a limited support for the effect of sunlight on reducing prostate cancer. Also, a study showed contradictory results that high levels of UVR exposure may be positively associated with the risk of prostate cancer mortality [116]. Another group of investigators [117, 118] used their ecological approach to conduct a multicountry study consisting of 33 countries worldwide to evaluate the effect of residential UV exposure on cancer incidence. The study results did not prove that sunlight/UV exposure could decrease the risk of various cancers including prostate cancer. The investigators of this study emphasized the importance of the control for various confounders that might have been overlooked in other studies.

## 5. Circulating Vitamin D and ProstateCancer Risk

Unlike most of sunlight exposure studies, linking circulating vitamin D levels or vitamin D uptakes with the reduction of prostate cancer risk has not been very successful. Of course, there are some studies [119–122] seeming to support the notion that high levels of serum vitamin D have protection effects against prostate cancer. A US study indicated that serum 1,25 vitamin D_3_ was negatively associated with prostate cancer restricted to men above median age of 57 years [119]. In a Fannish study with 13 yr followup of about 19,000 men, the authors found that low serum 25(OH)D_3_ concentrations were associated with high risk of earlier exposure to and more aggressive prostate cancer [120]. In addition, there are two more recent reports [121, 122] with an 18 yr or a 44.0 month median time followup, respectively, suggesting that both circulating 25(OH)D_3_ and 1,25(OH)_2_D_3_ or 25(OH)D_3_ alone at median or higher than medium levels have lower risk for prostate cancer progression. 

In fact, there are a good number of studies demonstrating no inverse relationship between circulating vitamin D metabolite levels and risk of prostate cancer [123–129]. For example, a very recent meta-analysis study [127] of relationship of serum 25-hydroxyvitamin D_3_ levels with colorectal, breast and prostate cancer and colonic adenoma was reported, showing, although there was a consistent inverse relationship between circulating vitamin D metabolite levels and colorectal cancer, no support for an association for breast and prostate cancer was found. Another recent nested prospective case-cohort study examined older men (65 or >) participating in the multicenter Osteoporotic Fractures in Men study for serum 25-OH vitamin D_3_ levels [128]. In this prospective cohort, the authors concluded that there was no association of serum 25-OH vitamin D_3_ levels with subsequent prostate cancer risk. A large nested case-control study [119] with a European population [129] indicated no beneficial effects of blood vitamin D levels for reducing prostate cancer risk. Another recent large prospective study [130] also did not show that vitamin D had effects on the reduction of prostate cancer risk. On the other hand, the same report stated that higher blood 25(OH)D_3_ levels could be related to increased risk of aggressive prostate cancer. A longitudinal nested case-control study was performed on Nordic men consisting of [131] 622 prostate cancer cases and 1,451 matched controls for serum 25(OH)D_3_ levels. Intriguingly, the study revealed a U-shaped relationship of prostate cancer risk and 25(OH)D_3_ levels, namely, both low (≤19 nmol/L) and high (≥80 nmol/L) 25(OH)D_3_ serum levels showed positive association with prostate cancer risk, whereas normal average serum concentrations of 25(OH)D_3_ (40–60 nmol/L) gave the lowest risk of prostate cancer. 

It may be worth mentioning that a nested case-control study [132] in the Health Professionals Follow-up Study was designed to determine the relationship of plasma 25(OH)D_3_ and 1,25(OH)_2_D_3_ with prostate cancer risk. Although it was found that there were no statistically significant differences between the two plasma vitamin D metabolites and the overall prostate cancer risk, a significant inverse association existed between 25-hydroxyvitamin D and advanced prostate cancer when comparing quintile 4 or quintile 5 to the bottom quintile. Moreover, when men who were clinically deficient either vitamin D metabolite compared to those who were not deficient, deficient group showed a 38% lower risk of total prostate cancer, a 58% lower risk of poorly differentiated prostate cancers, and a 49% lower risk of aggressive prostate cancers. An earlier study also reported that only older group (>61 years) with plasma 25(OH)D_3_ lower than the median showed a 57% reduction of cancer risk [133]. These unexpected results warrant further investigation.

Recent studies also could not find any association of vitamin D uptake with prostate cancer risk [134, 135]. With a mean followup of 8 years, examining men involved in the Multiethnic Cohort Study (1993–2002) using quantitative food frequency questionnaire revealed that there was no significant association between calcium and vitamin D intake and risk of prostate cancer. In the Prostate Cancer Prevention Trial (United States and Canada, 1994–2003) with 9,559 participants, dietary or supplemental intakes of vitamin D as well as many other factors analyzed did not show any significant correlation with prostate cancer risk. A meta-analysis of many observational studies regarding dairy products, calcium, and vitamin D intake and the risk of prostate cancer was also conducted [136]. This study concluded that there was no significant association of dietary vitamin D uptake with the cancer risk.

## 6. Genetic Variations in Vitamin D SignalingPathways and Prostate Cancer Risk

Since expression and functions of VDR, related vitamin D metabolic enzymes and vitamin D signal downstream genes are associated with vitamin D's action, genetic variation such as the single nucleotide polymorphisms (SNP) in these genes may have impact on vitamin D action in cancer cells. Therefore, analysis of the correlation of these polymorphisms with cancer risk would be highly meaningful. Among more than 470 polymorphisms in the VDR gene [11, 17], there may be six polymorphisms including the Fok1, Cdx2, Bsm1, Apa1, and Taq1 SNPs, and Poly(A) microsatellite to be frequently studied in relation to risk of cancer including prostate cancer.

The five SNPs mentioned above are usually detected by using restriction fragment length polymorphism or direct sequencing, and the PolyA microsatellite at the 3′-untranslated region (3′UTR) of the VDR gene is measured with variable number of tandem repeat. These polymorphisms are located in 5′regulatory, coding, intron, or 3′UTR of the VDR gene. The functionality of these polymorphisms used in epidemiological and observational studies for cancer risk may have been somewhat demonstrated by experimental approaches yet not completely resolved. For instance, Fok1, located in the exon 2, consists of a T to C change resulting in a longer protein translation (ff versus FF). The short VDR protein (i.e., 424 aa) encoded by the Fok1 FF genotype has a higher transcriptional activity than the long ff protein (i.e., 427 aa) [136–139]. Interestingly, it was also reported that individuals with ff genotype may be associated with lower serum 25(OH)D_3_ levels than those in individuals with FF genotype [140]. For Cdx2 polymorphism, it has a change from G to A in the binding site for an intestinal-specific transcription factor, CDX2, within the VDR promoter [141], the G allele of the Cdx-2 binding element has a significantly lower electrophoretic gel mobility shift assay activity and a lower transcription assay activity than that of the A allele. Although the PolyA microsatellite polymorphism has been suggested to be important for mRNA stability, studies show inconsistent conclusions, in which a report showed that the length of the UTR has no effect on mRNA stability [142], but other demonstrated that it has stability effects when interacting with Fok1 F allele [139]. Both Bsm1 and Apa1 SNPs are located in the intron near exon 9, and Taq1 SNP is located at exon 9 (which contains the 3′UTR). The potential function roles of these three SNPs in the regulation of VDR mRNA expression were examined briefly, but no conclusive results were produced [142–145]. 

Indeed, genetic heterogeneity effects of vitamin D signaling related genes, especially the VDR gene, have been attractive research subjects for cancer risk studies and potential applications in cancer prevention strategy. Although there are many of this type of epidemiological analyses [106, 146–172] as listed in Table 1, the apparent question is whether genetic variants of those genes involved in vitamin D pathways have real effects on prostate cancer risk. However, as shown in Table 1, the outcomes remain inconsistent and perhaps conflicting and require further studies.

## 7. Concluding Remarks

Although *in vivo* and *in vitro* laboratory studies provide strong evidence in supporting that vitamin D via VDR possesses antiprostate cancer activities, epidemiological studies have not shown consistent results for vitamin D's antiprostate cancer activities. Among many epidemiological studies, especially those studies with measuring blood vitamin D levels produced the least overall supporting evidence for the antiprostate cancer activities. One drawback of this type of studies is that the designs mainly relied on one measurement of serum/plasma vitamin D metabolites without multiple measurements in an adequate follow-up time. The conclusion from these epidemiologic studies for prostate cancer as well as other cancers are also reflected in the Institute of Medicine's 2011 report on dietary reference intakes for calcium and vitamin D [9] which could not make any conclusion if vitamin D has anticancer activities in humans. However, the inconsistency of outcomes of the epidemiologic studies may still provide a great deal of opportunities for further looking into and understanding very complexed vitamin D pathways for human cancer prevention. For example, some studies indicated that high level of serum vitamin D may, instead of decrease, increase risk of prostate cancer development or progression. The possible explanation seems to involve in local prostatic expression levels of the two vitamin D metabolizing enzymes, CYP27B1 and CYP24A1, as discussed above, which can be regulated by vitamin D, androgens and other dietary compounds. Potentially, overexpression of CYP24A1 could induce vitamin D resistance and promote risk for prostate cancer. Measuring serum vitamin D may not represent its levels at local tissues. Moreover, there is almost no information about the regulation and activities of these enzymes, as well as vitamin D metabolites in normal and cancerous prostate tissues under the *in vivo* conditions. Similarly, there is lacking of comprehensive information of *in vivo* VDR-mediated pathways in prostate cancer tissues. This could involve the interactions of genetic, epigenetic, and other endogenous and environmental factors at local tissue levels and will present challenges for developing more sophisticated study designs in the near future.

## Figures and Tables

**Table 1:** Summary of outcomes of 28 studies on the association of analysis of polymorphisms in vitamin D signal related genes with prostate cancer risk.

Gene(s)		Polymorphism(s)	Case/control	Results
(1)	VDR	Taq1	108/170	The tt genotype reduced cancer risk compared to the Tt or TT genotype [146]

(2)	VDR & AR	AR CAG repeat and VDR PolyA	57/169	The short CAG or long PolyA increased cancer risk and both increased risk of advanced cancer [147]

(3)	VDR	BsmI, ApaI, and Taq1	222/128 A Japanese population	Only BsmI BB or Bb genotype was associated with one-third of the risk of prostate cancer in a Japanese population [148]

(4)	VDR	BsmI, ApaI, and Taq1	81 familial cases/105 A Japanese population	No association [149]

(5)	VDR	Fok1, BsmI, Taq1, and PolyA	A meta-analysis of 14 studies	No association [150]

(6)	VDR	FokI, BsmI, ApaI, TaqI, and PolyA	African Americans (113/121) Whites (232/171)	No association but Fok1 FF genotype was associated with cancer risk in young African American [151]

(7)	VDR	Apal and Taql	165/200 A Brazilian population	No association [152]

(8)	VDR	BsmI, FokI, and PolyA	559/523	No association with overall cancer risk, but the BsmI bb was associated with a modest risk increase of localized prostate cancer compared to the BB genotype [153]

(9)	VDR	BsmI, ApaI, and TaqI	160/205 A Taiwanese population	No overall cancer risk association with ApaI and TaqI. But the Bsm1 BB and Bb versus the bb genotypes were negatively associated with risk of cancer [154]

(10)	VDR	BsmI and TaqI	428 white men and 310 African-American men	No association with overall cancer risk except that the Bsm1 B allele was inversely associated with recurrence of locally advanced cancer among white men [155]

(11)	VDR	CDX-2, Fok1, and Taq1	368 cancer/243 BPH	CDX-2 GA and AA and Fok1 ff were associated with increased prostate cancer risk in men with UVR exposure above the median, but genotype combinations such as GGTT and FFTT were associated with reduced risk in the higher UVR exposure group [106]

(12)	VDR	Cdx2, Fox1, Taq1, and Bgl II	450/455	FokI FF or Ff, TaqI tt, and BglI BB genotypes not Cdx2 genotypes may significantly protect against cancer progression with high sun exposure [156]

(13)	VDR	Fok1	128/147 An Indian population	Only the FF genotype showed an increased cancer risk [157]

(14)	VDR	FokI and BsmI	812/713 An Australian population	These two SNPs had no effect on prostate cancer risk [158]

(15)	VDR, VDBP	Novel sequence variations in the two promoters	165/324 African-American men	A novel VDR-5132 T/C SNP (i.e., CC genotype) was found to increase cancer risk in African-American men [159]

(16)	VDR	TaqI, BsmI, ApaI, FokI, and Poly(A)	A meta-analysis of 26 studies	No association [160]

(17)	VDR, CYP27A1, CYP24A1	38 SNPs	630/565	Only two VDR SNP loci, rs2107301, and rs2238135, with TT and CC genotypes, respectively, had a 2- to 2.5-fold increased risk of prostate cancer compared with the respective homozygote CC and TT alleles [161]

(18)	VDR	Fok1	1,066/1,618	The Fok1 ff genotype was found to increase cancer risk when 25(OH)D levels were lower than the median [162]

(19)	VDR	SNPs in haplotype block subregions C2 and C1	430 cancer/430 BPH UK men	Haplotype block C including G(3436)-A(3944)-C(20965)-C(30056), (G or C)-A-C-C, and G-A-(C or T)-C was found significantly to increase cancer risk in men with very low UVR exposure [163]

(20)	VDR	Apa I, BsmI, and Taq I	133/157 a Turkish population	Prostate cancer risk was found to be increased in those whose genotypes were either the Aa or aa compared to those with the AA type [164]

(21)	VDR, SRD5A2	Fox1 or Cdx2 and SRD5A2 V89L	444/488 Non-Hispanic White (NHW) men 141/273 Hispanic White (HW) men	Prostate cancer risk was increased by the interaction of the genotype with VDR SRD5A2 V89L VV *Fok*I TT/CT genotypes in NHW men and the interaction of the SRD5A2 V89L VV genotype with VDR CDX2 GG genotypes in HW men [165]

(22)	CYP27A1, GC, CYP27B1, CYP24A1, VDR, 7 vitamin D signaling downstream genes	212 SNPs	749/781	No association with overall cancer risk except that the BsmI and rs11574143 were associated with cancer risk only in men with lower 25(OH)D levels [166]

(23)	VDR	Fok1,Cdx2,Bsm1, Apa1, and Taq1	1,604 cases plus a meta-analysis of 13 studies	The BsmI (bb versus BB +Bb), ApaI (aa versus AA+Aa), and TaqI (Tt + tt versus TT) SNPs were determined to be associated with high Gleasone scores/cancer progression [167]

(24)	VDR		a meta-analysis of several cancers	For prostate cancer, Caucasian men with BsmI Bb would have significant reduction in cancer risk compared with bb genotype. It also concluded that Fok1 ff would contribute to the increase in cancer risk compared with FF genotype [168]

(25)	VDR	Taql, Apal, Bsml, Fok1, and CDX2	a meta-analysis of 36 studies	The Taql t and Bsml B alleles were found to be inversely associated with the risk. the Apal a allele was contributed to the reduction of cancer risk only in Asian populations, and the Fokl f allele was contributed to increased cancer risk only in Caucasian populations [169]

(26)	VDR, CYP27B1, CYP24A1	48 SNPs	827/787	No significant evidence of association of any of these SNPs (including VDR *Bsm*I, *Taq*I, *Apa*I and *Fok*I) with overall cancer risk or risk for tumor aggressiveness was found [170]

(27)	VDR	FokI, BsmI, Tru9I, ApaI, and TaqI	122/130 A Chinese Han population	Only BsmI B allele was found to be inversely associated with cancer risk compared with the b allele [171]

(28)	VDR, CYP19A1, CYP17A1, and AR	common SNPs in VDR, CYP17A1 CYP19A1 CAG repeat in AR	95 Italian heredofamilial prostate cancer (HFPC) cases/378 sporadic cancer	Only SNP rs10735810 (VDR1) T/T genotype in exon 4 of and SNP rs731236 (VDR2) T/T genotype in exon 11 of VDR showed positive interaction resulting in increased cancer risk for the HFPC patients compared to sporadic cancer [172]
